# How communities can control trachoma without a big budget

**Published:** 2012

**Authors:** Stephanie Ogden, Paul Emerson

**Affiliations:** WASH/NTD Coordinator, International Trachoma Initiative, Children Without Worms and Emory Center for Global Safe Water Email: sogden@taskforce.org; Director, Trachoma Control Programme, The Carter Center, Atlanta, USA Email: Paul.Emerson@emory.edu

Trachoma is an eye infection that affects an estimated 325 million people in Africa, Asia, and the Americas, and is the world's leading cause of preventable blindness. Infection occurs most readily in children, causing itching, redness, and irritation in the eyes and eyelids, and infected ocular discharge. Repeated infections in childhood lead to the formation of scar tissue which culminates in the inversion of the eyelids and eyelashes in adulthood, and ultimately, blindness.

Blindness from trachoma is preventable in every community in the world – right now. The World Health Organization (WHO) and its partners have set 2020 as the target to eliminate blinding trachoma globally, and communities can do this individually by breaking the cycle of infection and re-infection.

Trachoma control programmes have two major thrusts: providing surgery to patients at immediate risk of blindness from trachomatous trichiasis (see Comm Eye Health J 2012;25(78):38) and preventing transmission of the bacteria so that subsequent generations do not progress to the chronic, blinding stages of the disease.

The WHO endorses and promotes the SAFE strategy for trachoma control.

**Figure 1:**
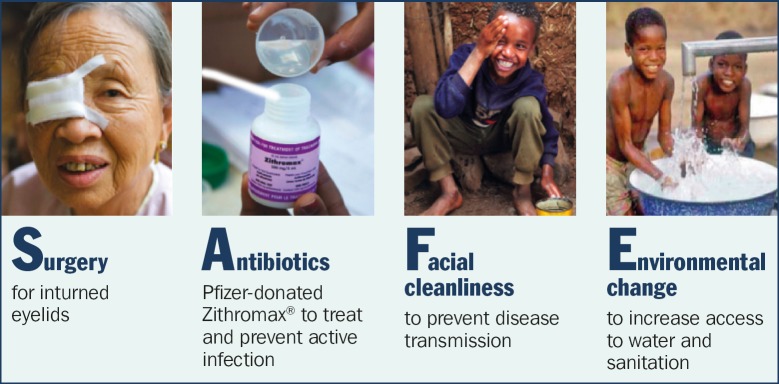
The SAFE way to controlling trachoma

While the S (surgery; Figure 1) of SAFE can repair in turned eyelids and prevent damage to the cornea, the A, F and E components can stop transmission of the disease – through treatment of current infections with antibiotics, facial cleanliness, and environmental change that encompasses the provision of water and sanitation. Many country programmes benefit from the generous donation of Zithromax® from Pfizer Inc. to address the A component, and donors provide funds for distribution. However, the F and E aspects of SAFE typically don't benefit from the same level of support, despite increasing evidence that halting trachoma transmission may not be possible without these vital activities that are often considered to be either too difficult or too expensive to implement.

**‘Individual and household behaviour are the key to trachoma prevention’**

The key to trachoma prevention is not complex infrastructure, but rather individual and household behaviour that prioritises and acts to ensure that faces are clean, that all household members dispose of their faeces in a safe way, and that households are free of material that attracts flies.

Facial cleanliness, hygiene promotion, and access to water and sanitation should be thought of as the cornerstones of trachoma prevention – to which antibiotics (the A component) can be added. The importance of the F component is two-fold: first, washing children's faces ensures that infectious eye and nose discharge that can be spread to others is washed away. Secondly, removing mucus, traces of food, and other material from children's faces decreases their attractiveness to eye-seeking flies that can carry the bacteria from one child's face to another. The E component, access to water and sanitation, changes the environment from one that favours transmission of trachoma to one which reduces it – and simultaneously contributes to the Millennium Development Goals, which call on each country to reduce by half the population of those without access to safe water and sanitation between 1990 and 2015. The E component also includes both household and wider environmental sanitation. In a practical sense, this means that all households should have access to a latrine (and that they use it), and that households and communities engage in proper waste management that limits the amount of human and animal faeces, food scraps, and excess moisture that attract flies.

Past implementation of the E component has largely focused on encouraging household sanitation and decreasing open defecation. The trachoma vector Musca sorbens is a fly that breeds in human faeces left on the ground out of the direct sun. However, it is not able to breed inside of latrines, where temperatures and moisture are typically too high and oxygen concentrations too low. Ensuring that households have access to and use a latrine for defecation helps to decrease the number of breeding grounds, and results in a reduced adult fly population within the community at large. Similarly, ensuring that communities engage in proper waste disposal, and that households and their adjacent compounds, as well as community areas such as schools, clinics, and streets are swept clean of animal waste and food scraps helps to decrease attractants to flies and reduce overall fly population.

Ensuring facial cleanliness and environmental sanitation is not just about expensive water projects. Facial and hand hygiene (with commercial or locally made soap if available) can be accomplished with small amounts of water used sparingly. Mothers of families have shown us that 1 litre of water can be sufficient to ensure that several children's faces are kept clean throughout the day. Programmes can celebrate existing positive hygiene practices in the communities, promote them as being achievable, and train community health agents to replicate them.

There are many steps to prevent trachoma that require little cost to households or to the wider community. Preventing trachoma starts with simple tasks.

**Tips for community health promoters**

Here's what you can do to help prevent trachoma:

- Promote face washing among families – especially children. Even small amounts of water can be used to clean children's faces throughout the day so that flies are not attracted to them.
- Teach families that sharing towels or cloths can put their loved ones at risk of infection if someone has trachoma. The bacterium that causes trachoma is easily spread through towels, bed sheets, clothes and wash cloths. These should be washed with soap to kill trachoma-causing bacteria.
- Ensure that trachoma prevention and hygiene education are taught in primary schools.
- Encourage every household in your community to maintain access to a latrine:
  – educate community members of the danger of open defecation. Even one open defecation site in the community may put all community members at risk for trachoma
    **Figure:**
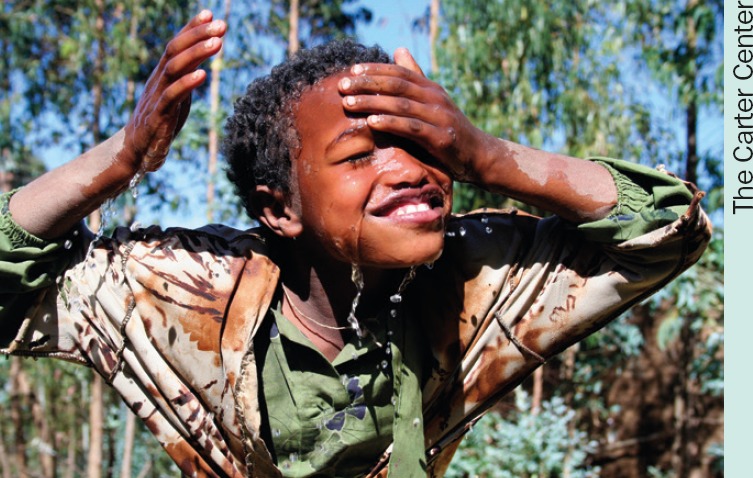
Encourage regular face washing
  – encourage community-wide cooperation and vigilance to encourage community wide sanitation
  – encourage households without a latrine to build, use, and maintain one
  – encourage households with a latrine to allow neighbours to use it until they have built their own
  – conduct a community household sanitation survey. Share this data with local health clinics, government offices or non-governmental organisations (NGOs) to help them understand the magnitude of the community's sanitation challenge.
- Encourage families to dispose of children's faeces safely. Faeces must be thrown into the latrine or buried to prevent flies from breeding in it. Children's faeces left in the open carry the same risk of spreading trachoma as open defecation.
- Congratulate families who sweep their compounds every day to keep them clean, and celebrate them as community role models. Clean compounds look good and decrease the number of flies that can carry trachoma from one face to another.
- Organise women's groups, village trachoma control committees, or school health and hygiene clubs to actively promote trachoma prevention.
- Make connections with local NGOs that work in water, sanitation and hygiene. Help them to target WASH improvements in communities where trachoma is highly prevalent.

**Case study: Village in Mali makes soap to fight trachoma**

Safia is a trachoma community health volunteer in Mali. She lives in the village of Sokoura which is 180 km from Mopti, the regional capital. Safia received training in how trachoma is transmitted, how it can be prevented, and the devastating effects that it can have in later life in a national training programme. She is a vibrant community member involved in soap production through a microfinance initiative supported by the programme and also provides regular, individual health education to women in her community. We spoke with her at home just after she had finished preparing a batch of local soap for sale in the market and asked about her involvement with the trachoma control programme.

**Figure:**
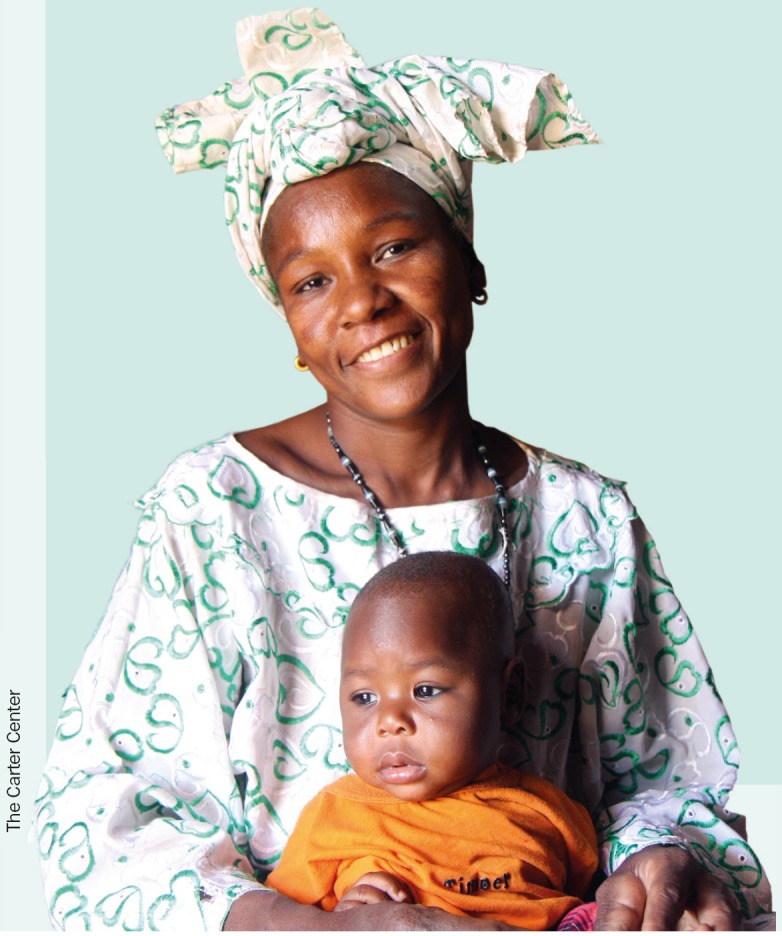
Safia's family gets free soap, and she can buy things for her children with the money she earns. MALI

“Once a year the national trachoma control programme organises a campaign to distribute medicine for trachoma. I work with my neighbours in the community to prevent trachoma transmission twelve months of the year. During my training, I was moved by the thought that I could help my community protect themselves from blindness, and I wanted to do what I can for my friends. I was already a member of the womens’ group, so it is easy for me to discuss new ideas and think about new ways of doing things. The womens’ groups have organised themselves to conduct village cleaning from time to time. We feel proud that the publicplaces are clean like our own compounds.

**Figure:**
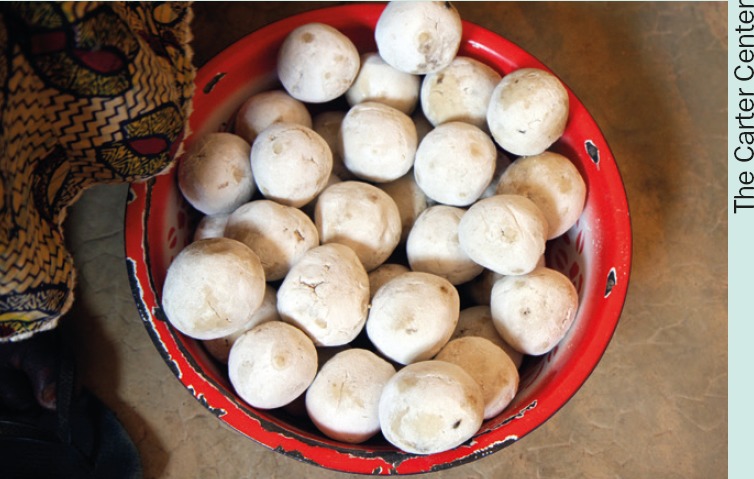
Women in a Mali village got a loan to produce and sell soap locally

**‘I work with my neighbors in the community to prevent trachoma transmission’**

“One of the best ideas that was given to us was microfinance for soap production. We make a loan of 5,000 CFA (approximately US $11) for the purchase of materials and the recipient pays back a total of 5,250 CFA from her profits. The soap production is very popular because it allows women to have soap for their own family for free and gives a little financial independence, so that we can buy treats for our children. The increased availability of soap should help everyone improve their hygiene and make laundry day easier.”

